# Supplementary material for: Causal Relationship between Aging and Anorexia Nervosa: A White-Matter-Microstructure-Mediated Mendelian Randomization Analysis
Source: Biomedicines. 2024 Aug 16;12(8):1874. doi: 10.3390/biomedicines12081874 (PMC11351342; doi:10.3390/biomedicines12081874)
Supplement: Supplementary file 1 [file biomedicines-12-01874-s001.zip › Supplementary Figure/Figure S9/Leaveoneout plot of MR analysis for ubm-b-1904 to AN.pdf]

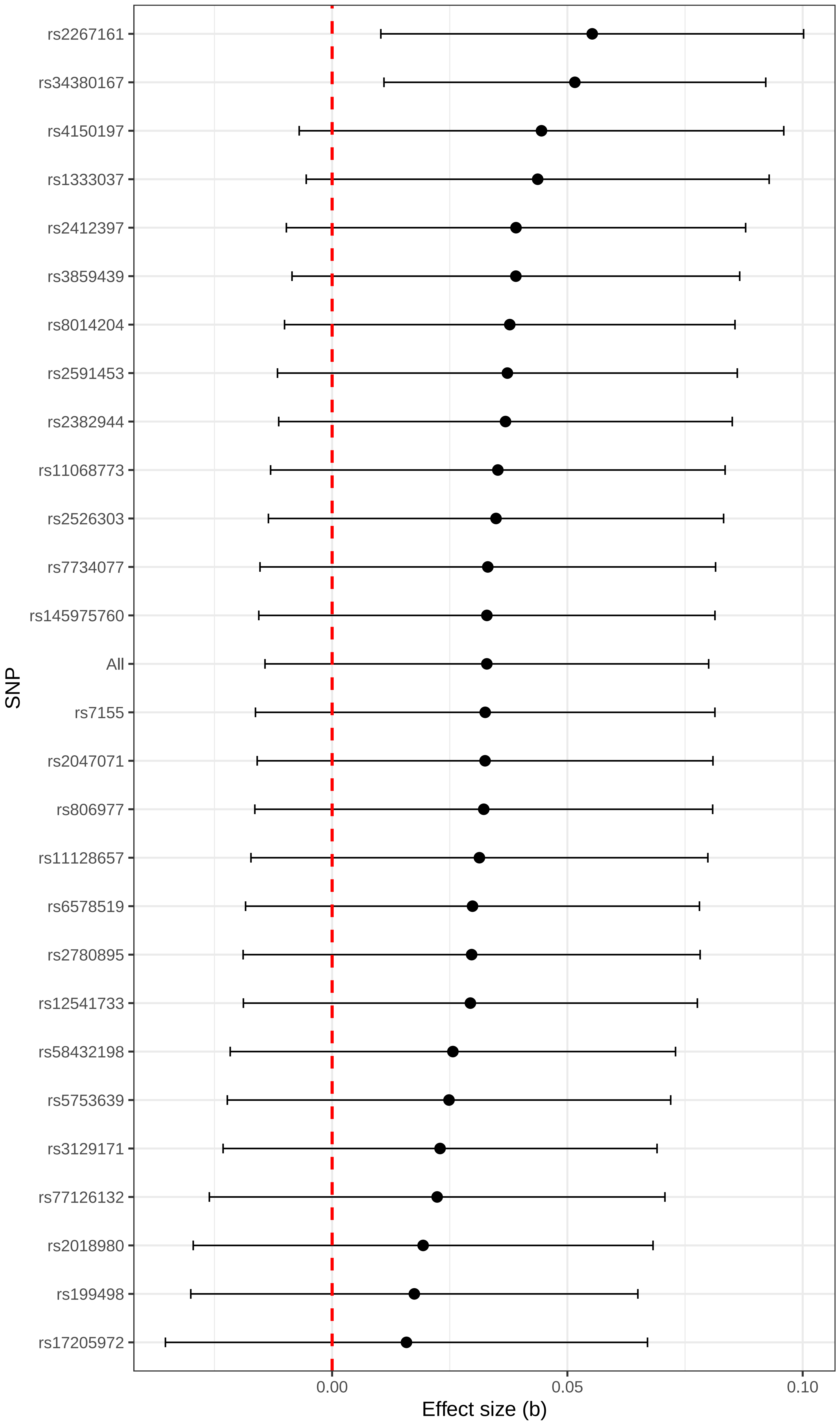
rs2267161
rs34380167
rs4150197
rs1333037
rs2412397
rs3859439
rs8014204
rs2591453
rs2382944
rs11068773
rs2526303
rs7734077
rs145975760
All
rs7155
rs2047071
rs806977
rs11128657
rs6578519
rs2780895
rs12541733
rs58432198
rs5753639
rs3129171
rs77126132
rs2018980
rs199498
rs17205972
SNP
0.00
0.05
0.10
Effect size (b)
